# Pituitary apoplexy in a pregnant woman

**DOI:** 10.4103/0972-2327.48861

**Published:** 2009

**Authors:** Vijay Parihar, Y. R. Yadav, Dhananjaya Sharma

**Affiliations:** Neurosurgery Unit, Department of General Surgery, NSCB Government Medical College and Hospital, Jabalpur, Madhya Pradesh - 482 002, India

**Figure 1:**
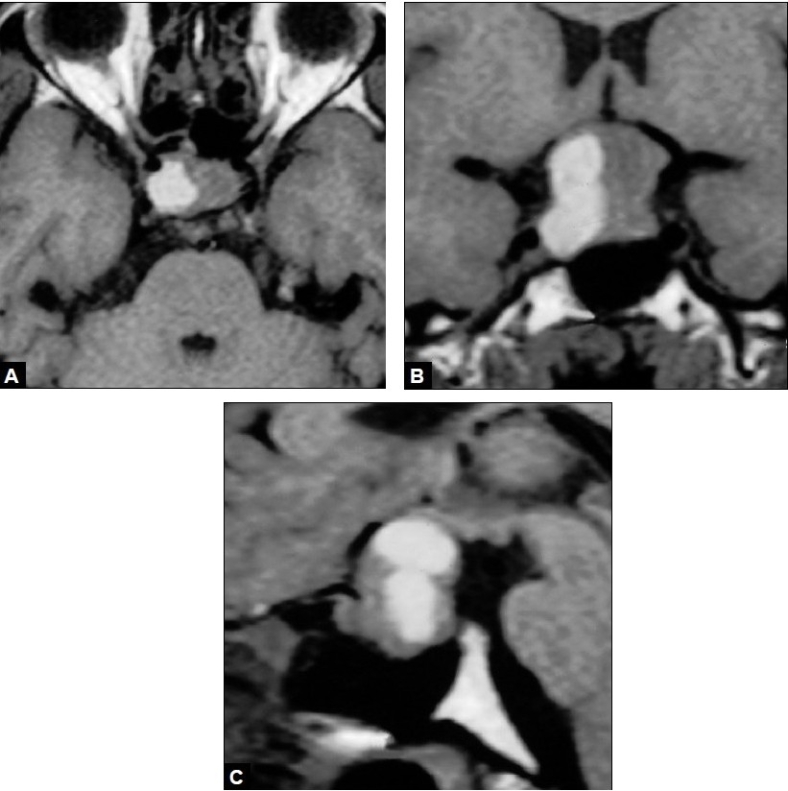
A: MRI Brain T1W (axial) images showing pituitary tumor with intratumoral hemorrhage; B:MRI Brain T1W (Coronal) images showing lelevation of optic chiasma; C: MRI Brain T1W (sagittal) images showing pituitary tumor with intra and suprasellar extension with pituitary aoplexy

A 22-year-old woman, a known case of macroprolactinoma, was under good control with bromocriptine therapy. Bromocriptine was stopped as pregnancy was diagnosed and serum prolactine level was <20 ng/ml. She was under regular neuroendocrinal and gynecological evaluation. In twentieth week of her pregnancy she presented with headache and vision loss. Rest of her CNS examination was normal. Serum prolactine level was 250.0 ng/ml. MRI of the brain showed pituitary apoplexy and compression over optic nerve and chiasma [Figure 1A–Figure 1C]. Ultrasonography of abdomen and pelvis was done and fetal well being was assessed. The patient underwent standard transsphenoidal decompression of gland and removal of hematoma. She had relief of headache and restoration of normal vision postoperatively. Histological examination showed prolactine cell adenoma hemorrhagic changes. Her serum prolactine level became normal (20.0 ng/ml). She gave birth to a full term healthy baby girl and had normal lactation. At the second year follow up she was doing well without any visual or neurological deficit.

MRI studies conducted in pregnant women have shown that the volume of pituitary gland may increase by 45% at a growth rate of 0.08 mm per week.[1] Pituitary adenoma may grow more rapidly in pregnant women. As a result tumor is at risk for hemorrhage.

Contrast-enhanced MRI is the accepted diagnostic method for pituitary adenoma, specifically for prolactinoma in patients presenting with elevated prolactin levels.[2–5] Signal intensity characteristics enable the differential diagnosis to be narrowed to a limited list of possibilities. These include neoplasms – craniopharyngioma, meningioma, germ cell tumor, epidermoid, dermoid, and Langerhans cell histiocytosis; aneurysms; congenital lesions – Rathke cleft cysts and arachnoid cysts; and infectious or inflammatory processes – tuberculosis, meningitis, sarcoidosis, and lymphocytic adenohypophysitis.[2,6] Proper differentiation is of clinical importance, because it will dictate the use of surgical versus nonsurgical treatments.[6]

A diagnostic distinction on MR images may be made between these sellar masses. Adenomas usually are isointense relative to gray matter on T1- and T2-weighted images.[2] Most tumors enhance intensely and homogeneously. Multilobulated margins are a reliable indicator of an adenoma. Osseous sellar-wall erosion, including extension into the sphenoid sinus or through the dorsum or tuberculum sellae, is typical. This erosion is less common in other masses involving the sellar and suprasellar regions. Displacement of arteries is more common than encasement, which is seen in only 19%  of reported macroadenomas.[4^8^] The infundibulum can be deviated laterally or upwardly displaced against the inferior hypothalamus.[2]

Compression of the optic nerves, tracts, and chiasma may be visualized, although large tumors may obscure these structures due to thinning and displacement. Contrast agent administration may be helpful in separating the optic structures from the tumor; however, it usually is unnecessary in evaluating the extent of the tumor because of contrast enhancement provided by the adjacent cerebrospinal fluid. Hemorrhage occurs in as many as 22% of all pituitary adenomas. An eccentric or central hyperintensity that corresponds to subacute hemorrhage, specifically methemoglobin, is visible on all MR images. Areas of cystic degeneration or necrosis, which have heterogeneous signal intensity characteristics, also may be observed in some macroadenomas. Cysts in necrotic adenomas are hyperintense on T2-weighted images and hypointense on T1-weighted images. Cysts do not enhance after contrast agent administration.[2]

In conclusion, MRI is the most helpful imaging study in these lesions to identify key distinguishing features helpful in making diagnosis leading to the proper approach and therapy.
